# Hepatic ^31^P‐magnetic resonance spectroscopy identified the impact of melatonin‐pretreated mitochondria in acute liver ischaemia‐reperfusion injury

**DOI:** 10.1111/jcmm.15617

**Published:** 2020-07-21

**Authors:** Sheung‐Fat Ko, Yi‐Ling Chen, Pei‐Hsun Sung, John Y. Chiang, Yi‐Ching Chu, Chung‐Cheng Huang, Chi‐Ruei Huang, Hon‐Kan Yip

**Affiliations:** ^1^ Department of Radiology Kaohsiung Chang Gung Memorial Hospital and Chang Gung University College of Medicine Kaohsiung Taiwan; ^2^ Division of Cardiology Department of Internal Medicine Kaohsiung Chang Gung Memorial Hospital and Chang Gung University College of Medicine Kaohsiung Taiwan; ^3^ Institute for Translational Research in Biomedicine Kaohsiung Chang Gung Memorial Hospital Kaohsiung Taiwan; ^4^ Center for Shockwave Medicine and Tissue Engineering Kaohsiung Chang Gung Memorial Hospital Kaohsiung Taiwan; ^5^ Department of Computer Science and Engineering National Sun Yat‐Sen University Kaohsiung Taiwan; ^6^ Department of Healthcare Administration and Medical Informatics Kaohsiung Medical University Kaohsiung Taiwan; ^7^ Department of Medical Research China Medical University Hospital China Medical University Taichung Taiwan; ^8^ Department of Nursing Asia University Taichung Taiwan; ^9^ Division of Cardiology Department of Internal Medicine Xiamen Chang Gung Hospital Xiamen China

**Keywords:** ^31^P‐magnetic resonance spectroscopy, liver ischaemia‐reperfusion injury, melatonin, mitochondria

## Abstract

Acute liver ischaemia‐reperfusion injury (IRI), commonly encountered during liver resection and transplantation surgery, is strongly associated with unfavourable clinical outcome. However, a prompt and accurate diagnosis and the treatment of this entity remain formidable challenges. This study tested the hypothesis that ^31^P‐magnetic resonance spectroscopy (^31^P‐MRS) findings could provide reliable living images to accurately identify the degree of acute liver IRI and melatonin‐pretreated mitochondria was an innovative treatment for protecting the liver from IRI in rat. Adult male SD rats were categorized into group 1 (sham‐operated control), group 2 (IRI only) and group 3 (IRI + melatonin [ie mitochondrial donor rat received intraperitoneal administration of melatonin] pretreated mitochondria [10 mg/per rat by portal vein]). By the end of study period at 72 hours, ^31^P‐MRS showed that, as compared with group 1, the hepatic levels of ATP and NADH were significantly lower in group 2 than in groups 1 and 3, and significantly lower in group 3 than in group 1. The liver protein expressions of mitochondrial‐electron‐transport‐chain complexes and mitochondrial integrity exhibited an identical pattern to ^31^P‐MRS finding. The protein expressions of oxidative stress, inflammatory, cellular stress signalling and mitochondrial‐damaged biomarkers displayed an opposite finding of ^31^P‐MRS, whereas the protein expressions of antioxidants were significantly progressively increased from groups 1 to 3. Microscopic findings showed that the fibrotic area/liver injury score and inflammatory and DNA‐damaged biomarkers exhibited an identical pattern of cellular stress signalling. Melatonin‐pretreated mitochondria effectively protected liver against IRI and ^31^P‐MRS was a reliable tool for measuring the mitochondrial/ATP consumption in living animals.

## 1 INTRODUCTION

Acute liver ischaemia‐reperfusion injury (IRI), which has been reported to commonly occur during liver resection and transplantation surgery is a crucial factor for predictive of poor outcome of liver transplantation.^1^, ^2^, ^3^ Studies have further displayed that several key factors contribute to the hepatic injury at the initiation and during the progression of liver IRI, including those of elevation of anaerobic metabolism, dysfunction of mitochondria, insult of oxidative stress, overload of intracellular calcium, activation of liver Kupffer cells, infiltration of immune cells and release of inflammatory cytokines.^2^, ^3^, ^4^, ^5^, ^6^ Although the underlying mechanisms of hepatic IRI have been extensively investigated, the clinical practice to prevent IRI is still limited. Additionally, despite the state‐of‐the‐art advance of critical care for patients with acute hepatic failure, the in‐hospital morbidity and mortality remain unacceptably high.^7^, ^8^, ^9^, ^10^, ^11^, ^12^ Therefore, it is of utmost importance to both clinician and medical researchers to explore a safe and efficacious therapeutic modality for patients with acute liver IRI refractory to conventional treatment.^13^, ^14^, ^15^, ^16^ However, prior to ascertain a novel, safe and efficacious therapeutic modality for acute liver IRI, the core property that induces this disease entity must be first clarified.

It is well recognized that mitochondria are especially rich in liver and heart. The function of mitochondria in liver or other organs not only serves as the primary energy source but also plays a pivotal role in extensive oxidative metabolism and normal function of the liver.^17^, ^18^ However, severely impaired mitochondrial function and activity have clearly identified in setting of liver ischaemia‐reperfusion^4^, ^19^, ^20^ with subsequent alternation in energy metabolism, generation of reactive oxygen species (ROS), inflammatory cell infiltration and cellular apoptosis^21^, ^22^, ^23^, ^24^ and ultimately irreversible damages to mitochondria,^16^, ^25^ exhausting ATP in ischaemia‐reperfusion liver.^4^, ^16^, ^21^, ^25^, ^26^, ^27^ These findings^4^, ^16^, ^19^, ^20^, ^21^, ^22^, ^23^, ^24^, ^25^, ^26^, ^27^ highlight that the quantity (ie amount) and quality (functional integrity) of mitochondria play extremely important roles on acute liver IRI. Accordingly, these aforementioned issues^4^, ^16^, ^21^, ^25^, ^26^, ^27^ raise the hypothesis that restoration of mitochondrial function through exogenous mitochondrial transfusion may be a potential strategy for the treatment of acute hepatic IRI.

Melatonin, acting as a scavenger of free radicals for cell membrane stabilization, has been identified to have powerful ancient antioxidant^28^ for suppressing the generation of oxidative stress.^29^ Basic researches have exhibited that melatonin enters mitochondria through oligopeptide transporters and specifically targets at the mitochondria where it seems to function as an apex antioxidant.^30^ In addition to cell uptake from circulation, melatonin is produced in the mitochondria as well.^30^ It is well recognized that oxidative damage is a result of free radicals produced in cells, especially in mitochondria.^31^ Recent study has further demonstrated the protective stabilization of mitochondrial permeability transition and mitochondrial oxidation during mitochondrial Ca^2+^ stress by melatonin's cascade metabolites.^32^ Thus, melatonin always plays a crucial role on protecting the mitochondrial number and functional integrity^30^, ^31^, ^32^ through regulation of the oxidative stress and the preservation of ATP/energy, resulting in protection of organs from IRI.^16^, ^30^, ^33^, ^34^, ^35^, ^36^

Interestingly, previous studies^37^, ^38^ have shown that energy metabolic rate could be detected by magnetic resonance imaging (MRI) study. Additionally, it is well known that liver organ which is an important vital organ contains abundant ATP to provide the adequate energy for metabolism and for storages of nutrient and cholesterol as well as for detoxication of drug and chemicals. Thus, the activity of liver could be non‐invasively identified by the phosphate, that is the intermediate side product of ATP. On the other hand, studies have further demonstrated that hepatic ^31^P‐magnetic resonance spectroscopy (^31^P‐MRS) is a valuable tool for detecting the metabolism of different liver disease entities.^39^, ^40^, ^41^ Thus, ^31^P‐MRS has advantages of non‐invasive and precise detection of active energy metabolic rate and energy storage (ie ATP level) in cells/organs such as hepatocytes/liver. Based on the aforementioned issues,^16^, ^17^, ^21^, ^25^, ^28^, ^29^, ^30^, ^33^, ^34^, ^35^, ^36^ this study tested the hypothesis that melatonin‐pretreated exogenous mitochondria might offer an added benefit on protecting the liver against acute IRI. Additionally, this study further tested whether the ^31^P‐MRS examination could provide reliable living images to accurately identify the degree of ATP consumption/depletion in hepatocytes, that is an indicator of acute liver ischaemia‐reperfusion in rodent.

## 2 MATERIALS AND METHODS

### 2.1 Ethics

All animal procedures were approved by the Institute of Animal Care and Use Committee at Kaohsiung Chang Gung Memorial Hospital (Affidavit of Approval of Animal Use Protocol No. 2018030802) and performed in accordance with the Guide for the Care and Use of Laboratory Animals.

Animals were housed in an Association for Assessment and Accreditation of Laboratory Animal Care International (AAALAC; Frederick, MD, USA)‐approved animal facility in our hospital with controlled temperature and light cycles (24°C and 12/12 light cycle).

### 2.2 Liver ischaemia‐reperfusion procedure, animal grouping and treatment strategy

The procedure and protocol have been described in our previous report^42^ and were based on the other previous studies.^43^, ^44^ In detail, the pathogen‐free, adult male Sprague Dawley (SD) rats (n = 24) weighing 325‐350 g were randomly divided into three groups: group1 (sham‐operated control [SC], laparotomy only with 500 μL of saline by portal vein administration), group 2 (IRI only, left liver lobe ischaemia for 60 minutes followed by reperfusion for 72 hours plus saline [500 μL/rat] from portal vein administration at the beginning of reperfusion) and group 3 (IRI plus melatonin‐pretreated mitochondria [Mito; 10 mg each rat] by portal vein administration at the beginning of reperfusion). The dosage of mitochondria utilized in the present study was based on our previous studies with some modifications.^42^, ^45^, ^46^

The procedure was described as follows: the left lobe of the liver was dissected free from the surrounding ligaments. Hepatic ischaemia was induced by temporal obstruction of the vessels by placing a 4‐0 silk loop around the hilar region of the left liver lobe in groups 2 and 3 animals, whereas SC animals received only laparotomy without undergoing hepatic ischaemia. Reperfusion was started 60 minutes later when the hilar occlusion was released.

All animals were killed by 72 hours after reperfusion. To elucidate the impact of acute liver ischaemia‐reperfusion on hepatocyte damage and up‐regulation of inflammatory reaction as well as the therapeutic effect of melatonin on the circulatory parameters, blood samples were collected before and after the experiments and plasma specimens were harvested for analyses of aspartate aminotransferase (AST) concentration, alanine aminotransferase (ALT) and circulating levels of inflammatory cytokines. Liver specimens were acquired and then stored at −80°C for individual study. Tissues were also embedded in optimal cutting temperature compound or 4% buffered formaldehyde for cryo‐sectioning and paraffin sectioning, respectively.

### 2.3 Melatonin therapy prior to mitochondrial isolation from donors, the procedure and protocol of mitochondrial isolation and MitoTracker staining for mitochondria

For stabilization and attenuation of oxidative stress in isolated mitochondria, melatonin was given to donor rat (n = 3) at day 3 (50 mg), 2 (25 mg) and 1 (25 mg)/rat by intraperitoneal administration prior to acute liver ischaemia‐reperfusion procedure.^16^, ^42^ The procedure and protocol of liver mitochondrial isolation from donor SD rats have been previously described by our studies.^42^, ^45^, ^46^ In detail, the rats (ie the donors) were fasted overnight prior to the mitochondrial isolation procedure, then killed and their gallbladders and livers carefully isolated and removed. Immediately, the liver (3 g) was immersed in 50 mL of ice‐cold IBc (10 mmol/L Tris–MOPS, 5 mmol/L EGTA/Tris and 200 mmol/L sucrose, pH 7.4) in a beaker, followed by rinsing the liver free of blood with ice‐cold IBc. The liver was then minced with scissors in a beaker surrounded by ice. IBc was discarded during mincing and replaced with 18 mL of ice‐cold fresh IBc. The liver was then homogenized with a Teflon pestle. The homogenates were transferred to a 50‐mL polypropylene Falcon tube and centrifuged at 600 *g* for 10 minutes at 4°C. The supernatants were transferred to fresh tubes for centrifugation at 7000 *g* for 10 minutes at 4°C. The supernatants were discarded, and the pellets washed with 5 mL ice‐cold IBc. Again, the supernatants from the pellets were centrifuged at 7000 *g* for 10 minutes at 4°C. The supernatants were discarded and the pellets containing the mitochondria resuspended. The concentration of the mitochondrial suspensions was measured using the Biuret method. Each 10 mg of isolated mitochondria was labelled with 1 mol/L of MitoTracker Red CMXRos (Invitrogen, Carlsbad, CA, USA) through incubation at 37°C for 30 minutes for tracing the mitochondria in the ischaemia‐reperfusion liver. Mitochondrial transfusion was performed for the study animals immediately after labelling (ie <3 hours after the isolation procedure).

### 2.4 Hepatic magnetic resonance imaging assessment

The liver MRI examination was performed using a 9.4‐T horizontal‐bore animal MR scanning system (Biospec 94/20; Bruker, Ettingen, Germany). This scanning system comprises a self‐shielded magnet with a 20 cm clear bore and a BGA‐12S gradient insert (inner diameter: 12 cm) that offers a maximal gradient strength of 675 mT/m. After adequate sedation by mask inhalation of isoflurane (2%), the rats were kept in shallow breathing and placed in a plastic holder in a prone position. For ^31^P‐MRS, a double‐tuned (^1^H/^31^P) surface coil (20 mm in diameter) tuned to 400.3 and 161.9 MHz was used for proton imaging and phosphorus spectroscopy covering whole liver region of rat.

### 2.5 ^31^P‐magnetic resonance spectroscopy

Serial ^31^P‐MRS examinations were performed on each rat at baseline (preoperatively), before‐surgery and day 1, 2 and 3 post‐surgery. The ^31^P‐MRS was acquired using a simple pulse‐acquisition sequence, with a pulse optimized to give a 90° excitation in the liver region with the added advantage of overtripping signal from the abdominal region. Respiratory gating was not used as the spectra acquired were nonlocalized, and hence, the resulting spectrum was the average ^31^P‐MRS signal from the sensitive area of the coil, which was optimized to have the largest signal (90° pulse) in the liver. The ^31^P‐MRS data were collected with a TR of 3000 ms, 512 averages, and a spectral width of 8000 Hz. Data were analysed in the time cross via NMR data processing guide of ParaVision5.1 (Bruker). The phosphomonoesters (PME), inorganic phosphate (Pi), phosphodiester (PDE), NADH and the three‐nucleotide triphosphate (mainly adenosine triphosphate) peaks in the spectrum were fitted after application of 0.5‐Hz line broadening and manual phasing.

### 2.6 Histopathological analysis for quantification of liver injury score and liver fibrosis at 72 hours

After preparation of haematoxylin and eosin (HE) stain, the degree of liver injury was determined according to our previous report with liver injury score defined as follows: 0 indicated no notable hepatocyte integrity impairment or sinusoidal distortion; 1 corresponded to mild hepatic injury with less than 25% of section involved; 2 indicated moderate hepatic injury with 25%‐50% of section involved; 3 denoted severe hepatic injury with more than 50% involved.^42^ For each animal, three liver sections were examined and three randomly selected high‐power fields (HPFs) (ie 200×) were analysed in each section. The mean number per HPF for each animal was then determined by summation of all numbers divided by 9.

Additionally, Masson's trichrome staining was utilized for investigating the fibrosis in liver parenchyma. Three serial sections of liver from each animal were prepared at 3 µm thickness by microtome (Leica RM2235, Buffalo Grove, IL, USA). The integrated area (µm^2^) of fibrotic area on each section was calculated using the Image Tool 3 (IT3) image analysis software (University of Texas, Health Science Center, San Antonio, UTHSCSA; Image Tool for Windows, version 3.0, USA). Three randomly selected high‐power fields (HPFs) (100×) were analysed in each section. The numbers of pixels in fibrotic area obtained from three HPFs were summated. The procedure was repeated in two other sections for each animal. The mean pixel number per HPF for each animal was then determined by summating all pixel numbers and divided by 9. The mean integrated area (µm^2^) of fibrosis in liver per HPF was obtained using a conversion factor of 19.24 (1 µm^2^ represented 19.24 pixels).^42^

### 2.7 Immunohistochemistry and immunofluorescence staining

The procedure and protocol of staining were based on our previous reports.^42^, ^45^, ^46^ In detail, rehydrated paraffin sections (3 µm in thickness) were first treated with 3% H_2_O_2_ for 30 minutes and incubated with Immuno‐Block reagent (BioSB, Santa Barbara, CA, USA) for 30 minutes at room temperature. Immunohistochemistry (IHC) staining was performed with MMP‐9 (1:200; Thermo Fisher Scientific, Waltham, MA, USA) primary antibody at room temperature for 1 hour. After addition of the anti‐mouse HRP‐conjugated secondary antibody, 3,3′‐diaminobenzidine (DAB) (Sigma‐Aldrich, St. Louis, MO, USA) was used to detect the signals. Sections were counterstained with haematoxylin as a counter‐stain for nuclei and observed with a light microscope (Olympus IX‐40). For immunofluorescence (IF) staining, cryostat sections (3 µm) were then incubated with primary antibodies specifically against γ‐H2AX (1:500; Abcam, Cambridge, UK), CD14 (1:200; Bioss Antibodies, Woburn, MA, USA) and CD68 (1:100; Abcam) at 4°C overnight, while sections incubated with the use of irrelevant antibodies served as controls. Alexa Fluor488‐conjugated goat anti‐mouse or rabbit IgG were used to localize signals. Sections were finally counterstained with DAPI and observed with a fluorescent microscope (Olympus IX‐40, Melville, NY, USA). Three sections of liver specimen from each rat were analysed. For quantification, three randomly selected HPFs (200× or 400× of IHC and IF microscopic findings) were analysed in each section. The mean number of positively stained cells per HPF for each animal was then determined by summation of all numbers divided by 9.

### 2.8 Western blot analysis

The procedure and protocol for Western blot analysis have been described in detail in our previous studies.^42^, ^45^, ^46^ Briefly, equal amounts (50 µg) of protein extracts were loaded and separated by SDS‐PAGE using acrylamide gradients. After electrophoresis, the separated proteins were transferred to a polyvinylidene difluoride (PVDF) membrane (Amersham Biosciences, Amersham, UK). Nonspecific sites were blocked by incubation of the membrane in blocking buffer (5% nonfat dry milk in T‐TBS [TBS containing 0.05% Tween 20]) overnight. The membranes were incubated with the indicated primary antibodies (complex I [1:2000; Abcam], complex II [1:2000; Abcam], complex III [1:2000; Abcam], complex V [1:2000; Abcam], dynamin‐related protein 1 [DRP1, 1:1000; Cell Signaling, Danvers, MA, USA], cyclophilin D [1:3000; Abcam], cytosolic cytochrome C [1:1000; BD Biosciences, San Jose, CA, USA], mitochondrial cytochrome C [1:1000; BD Biosciences], tumour necrosis factor [TNF]‐α [1:1000; Cell Signalling, su300; Abcam], matrix metalloproteinase [MMP]‐2 [1:1000; Cell Signalling], MMP‐9 [1:2000; Abcam], interleukin [IL]‐1 [1:1000; Cell Signalling], NOX‐1 [1:1500; Sigma‐Aldrich], NOX‐2 [1:750; Sigma‐Aldrich], haeme oxygenase [HO]‐1 [1:500; Calbiochem, San Diego, CA, USA], NAD(P)H dehydrogenase [quinone] 1 [NQO 1; 1:1000; Abcam], nuclear factor erythroid 2‐related factor 2 [(Nrf2; 1:1000; Abcam], phosphoinositide 3‐kinases [PI3K; 1:5000; Abcam], phosphorylated mammalian target of rapamycin [p‐mTOR; 1:1000; Cell Signalling], p‐Akt [1:1000; Cell Signalling] and beta‐actin [1:10 000; Chemicon, Billerica, MA, USA]) for 1 hour at room temperature. Horseradish peroxidase‐conjugated anti‐rabbit immunoglobulin IgG (1:2000; Cell Signalling) was used as a secondary antibody for 1‐hour incubation at room temperature. The washing procedure was repeated eight times within 1 hour. Immunoreactive bands were visualized by enhanced chemiluminescence (ECL; Amersham Biosciences) and exposed to Biomax L film (Kodak, Rochester, NY, USA). For the purpose of quantification, ECL signals were digitized using Labwork software (UVP™ BioSpectrum™ Imaging System, Upland, CA, USA).

### 2.9 Laboratory assessment of circulating levels of inflammatory biomarkers and liver function

Circulating levels of tumour necrosis factor (TNF)‐α, interleukin (IL)‐6 and myeloperoxidase (MPO), three indicators of soluble inflammatory biomarkers, were measured by duplicated determinations with a commercially available ELISA method (R&D Systems, Minneapolis, MN, USA). Intra‐observer variability of the measurements was also assessed, and the mean intra‐assay coefficients of variance were all <3.2%. Additionally, the concentrations of serum AST and ALT were measured by standard method in the Animal Laboratory Clinic.

### 2.10 Statistical analysis

Quantitative data were expressed as mean ± SD. Statistical analysis was adequately performed by ANOVA followed by Bonferroni's multiple comparison post hoc test. SAS statistical software for Windows version 8.2 (SAS Institute, Arlington, VA, USA) was utilized. A probability value <0.05 was considered statistically significant.

## 3 RESULTS

### 3.1 Circulatory levels of liver enzyme and inflammatory biomarkers by 72 hours after liver ischaemia‐reperfusion induction

**FIGURE 1:**
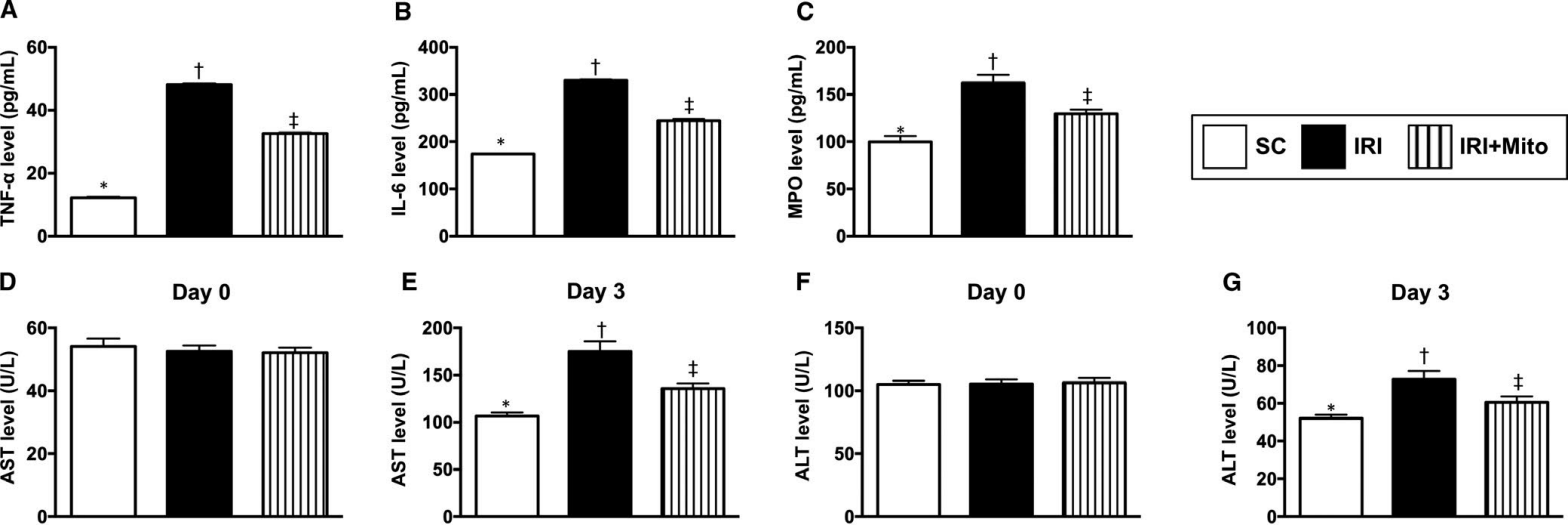
Circulatory levels of liver enzyme and inflammatory biomarkers by day 0 and 3 after liver ischaemia‐reperfusion injury induction. A, ELISA result of circulating level of tumour necrosis factor (TNF)‐α, * vs other groups with different symbols (†, ‡), *P* < 0.0001. B, ELISA result of circulating level of interleukin (IL)‐6, * vs other groups with different symbols (†, ‡), *P* < 0.0001. C, ELISA result of circulating level of myeloperoxidase (MPO), * vs other groups with different symbols (†, ‡), *P* < 0.0001. D, Serum level of aspartate aminotransferase (AST) at day 0, *P* = 1.0. E, Serum level of AST at day 3, * vs other groups with different symbols (†, ‡), *P* < 0.0001. F, Serum level of alanine Aminotransferase (ALT) at day 0, *P* = 1.0. G, Serum level of ALT at day 3, * vs other groups with different symbols (†, ‡), *P* < 0.001. All statistical analyses were performed by one‐way ANOVA, followed by Bonferroni's multiple comparison post hoc test (n = 8 for each group). Symbols (*, †, ‡) indicate significance (at 0.05 level). IRI, ischaemia‐reperfusion injury; Mito, mitochondria; SC, sham control

First, to elucidate how the circulating levels of inflammatory biomarkers and the liver enzymes were augmented after ischaemia‐reperfusion procedure, the ELISA method was utilized for examination of these parameters. The result demonstrated that by 72 hours after ischaemia‐reperfusion procedure, circulating levels of TNF‐α, IL‐6 and MPO, three indicators of inflammation, were significantly increased in IRI than in SC and IRI + Mito and significantly increased in IRI + Mito than in SC. Consistently, serum levels of AST and ALT, two indices of hepatocyte integrity, showed an identical pattern compared with that of inflammation. These findings imply that melatonin‐pretreated mitochondria therapy attenuated circulatory inflammatory markers and protected the liver from acute IRI (Figure 1).

### 3.2 MRI findings of hepatic phosphorylated metabolism by 72 hours after liver ischaemia‐reperfusion induction

**FIGURE 2:**
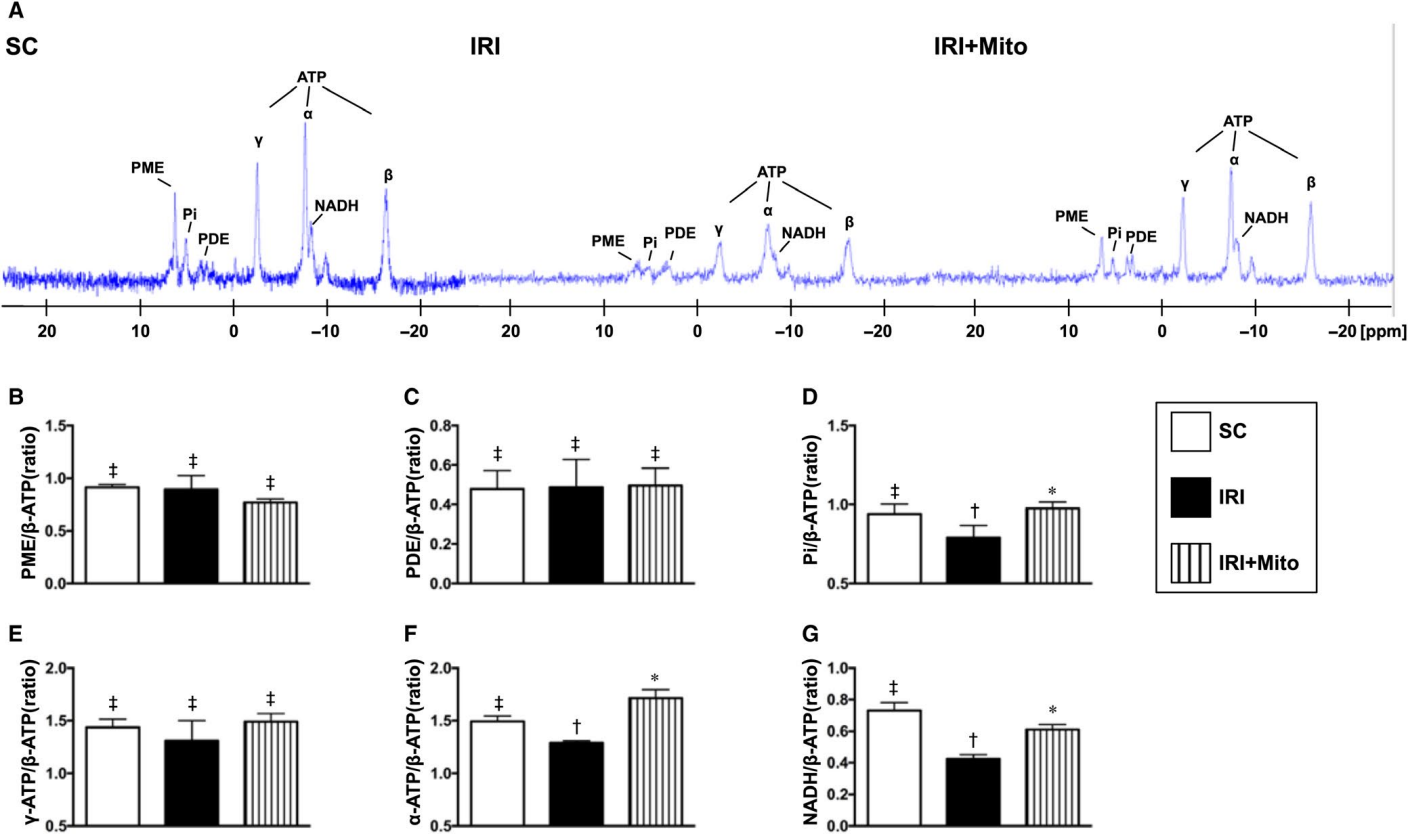
MRI findings of hepatic phosphorylated metabolism by 72 h after liver ischaemia‐reperfusion injury induction. A, Illustrating the hepatic ^31^P‐magnetic resonance spectra (ie ^31^P‐MRS) examination for identifying the ATP (an indicator of energy storage) in hepatocytes of SC, IRI and IRI + Mito animals, respectively. B‐G, The relative level of each metabolites was subtracted from the β‐ATP value. The data are expressed as mean ± SD (n = 8 per group). One‐way ANOVA followed by post hoc Tukey‐Kramer test was used for statistical analysis. Symbols (*, †, ‡) indicate significant differences (at 0.05 level). ATP, adenosine triphosphate; IRI, ischaemia‐reperfusion injury; NADH, nicotinamide adenine dinucleotide; PDE, phosphodiester; Pi, inorganic phosphate; PME, phosphomonoesters; SC, sham control

Next, to clarify whether MRI was a sensitive and reliable non‐invasive tool for determining the degree of liver IRI, the Phosphorus MR spectroscopy (^31^P‐MRS) was used in the present study. As expected, the result showed that as compared with SC, the hepatic levels of Pi, α‐ATP and NADH (ie complex V for generation of ATP) were substantially reduced in IRI than in SC and IRI + Mito, and significantly reduced in IRI + Mito than SC. However, γ‐ATP level was only relatively reduced in IRI as compared with the other two groups but it did not reach statistical significance, whereas the parameters of PME and PDE did not differ among the three groups. These findings suggested that exogenous mitochondrial administration enriched the hepatocyte mitochondria (Figure 2).

### 3.3 The protein expressions of mitochondrial integrity, mitochondrial‐electron‐transport‐chain complexes, oxidative stress and inflammatory biomarkers by 72 hours after liver ischaemia‐reperfusion induction

**FIGURE 3:**
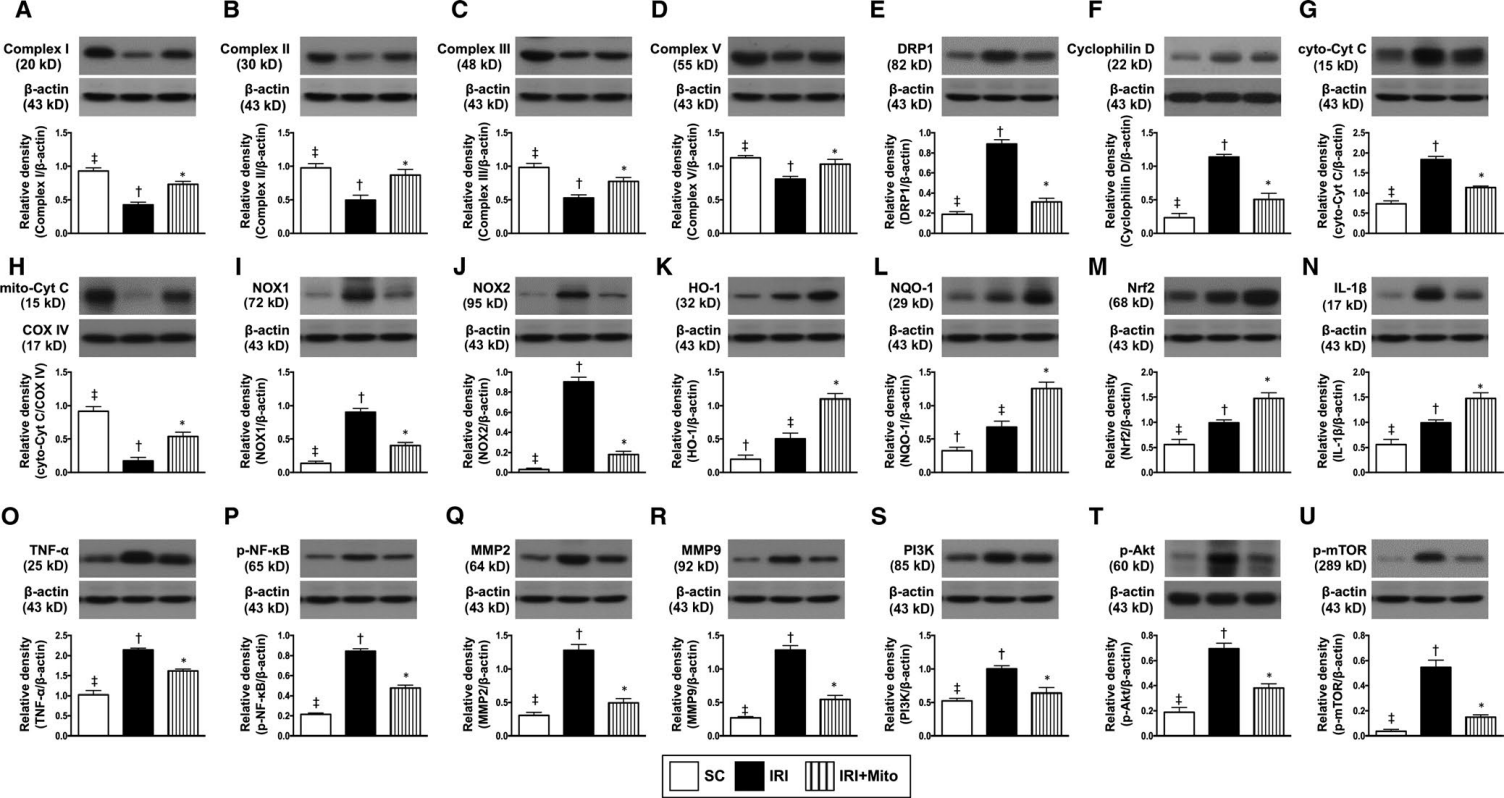
Protein expressions of mitochondrial integrity, oxidative phosphorylation, oxidative stress, inflammation and cellular stress signalling by 72 h after liver ischaemia‐reperfusion injury induction. A, Protein expression of complex I, * vs other groups with different symbols (†, ‡), *P* < 0.0001. B, Protein expression of complex II, * vs other groups with different symbols (†, ‡), *P* < 0.001. C, Protein expression of complex III, * vs other groups with different symbols (†, ‡), *P* < 0.0001. D, Protein expression of complex V, * vs other groups with different symbols (†, ‡), *P* < 0.001. E, Protein expression of dynamin‐related protein 1 (DRP1), * vs other groups with different symbols (†, ‡), *P* < 0.0001. F, Protein expression of cyclophilin (Cyc‐D), * vs other groups with different symbols (†, ‡), *P* < 0.0001. G, Protein expression of cytosolic cytochrome C (cyto‐Cyt C), * vs other groups with different symbols (†, ‡), *P* < 0.0001. H, Protein expression of mitochondrial cytochrome C (mito‐Cyt C), * vs other groups with different symbols (†, ‡), *P* < 0.0001. I, Protein expression of NOX‐1, * vs other groups with different symbols (†, ‡), *P* < 0.0001. J, Protein expression of NOX‐2, * vs other groups with different symbols (†, ‡), *P* < 0.0001. K, Protein expression of haeme oxygenase (HO)‐1, * vs other groups with different symbols (†, ‡), *P* < 0.0001. L, Protein expression of NAD(P)H dehydrogenase (quinone) 1 (NQO1), * vs other groups with different symbols (†, ‡), *P* < 0.001. M, Protein expression of nuclear factor erythroid 2‐related factor 2 (Nrf2), * vs other groups with different symbols (†, ‡), *P* < 0.01. N, Protein expression of interleukin (IL)‐1β, * vs other groups with different symbols (†, ‡), *P* < 0.0001. O, Protein expression of tumour necrosis factor (TNF)‐α, * vs other groups with different symbols (†, ‡), *P* < 0.0001. P, Protein expression of phosphorylated (p)‐nuclear factor (p‐NF)‐κB, * vs other groups with different symbols (†, ‡), *P* < 0.0001. Q, Protein expression of matrix metalloproteinase (MMP)2, * vs other groups with different symbols (†, ‡), *P* < 0.0001. R, Protein expression of MMP9, * vs other groups with different symbols (†, ‡), *P* < 0.0001. S, Protein expression of PI3K, * vs other groups with different symbols (†, ‡), *P* < 0.0001. T, Protein expression of p‐Akt, * vs other groups with different symbols (†, ‡), *P* < 0.0001. U, Phosphorylated mammalian target of rapamycin (p‐mTOR), * vs other groups with different symbols (†, ‡), *P* < 0.0001. All statistical analyses were performed by one‐way ANOVA, followed by Bonferroni multiple comparison post hoc test (n = 6 for each group). Symbols (*, †, ‡) indicate significance (at 0.05 level). IRI, ischaemia‐reperfusion injury; Mito, mitochondria; SC, sham control

It is well known that mitochondrial integrity and the stability of mitochondrial‐electron‐transport‐chain complexes (METCC) are fundamentally important for an effective‐energy generation of ATP in the cells. We therefore measured the protein expressions related to these two cardinal factors. Consistently, the protein expressions of cytosolic cytochrome C, DRP1 and cyclophilin D, three indices of mitochondrial‐damaged biomarkers, were significantly higher in IRI than in SC and IRI + Mito, and significantly higher in IRI + Mito than in SC, suggesting IRI depleted hepatocyte endogenous mitochondria. On the other hand, the protein expressions of complex I, II, III and V, four indictors of METCC, and mitochondrial cytochrome C, an index of mitochondrial integrity, were significantly higher in SC than in IRI and IRI + Mito, and significantly higher in IRI + Mito than in IRI, suggesting that exogenous mitochondrial transfusion enriched the mitochondria in the injured hepatocytes (Figure 3A‐H).

We further identified that the protein expressions of NOX‐1 and NOX‐2, two indicators of oxidative stress, were significantly increased in IRI than in SC and IRI + Mito and significantly increased in IRI + Mito than in SC. On the other hand, the protein expressions of HO‐1, NQO1 and Nfr2, three indicators of antioxidants, were significantly progressively increased from groups 1 to 3, suggesting an intrinsic response to ischaemia‐reperfusion stimulation that was enhanced by exogenous Mito therapy (Figure 3I‐M).

Molecular level of inflammatory reaction has been crystal clearly identified to be augmented in setting of acute ischaemia‐reperfusion.^16^, ^33^, ^36^ Here, we also found that the protein expressions of IL‐1ß, TNF‐α, p‐NF‐κB, MMP‐2 and MMP‐9, five indices of inflammation, were significantly increased in IRI than in SC and IRI + Mito, and significantly increased in IRI + Mito than in SC. Additionally, the protein expressions of PI3K, p‐Akt and p‐mTOR, three biomarkers of cellular stress signalling, displayed an identical pattern of inflammation among the three groups, suggesting that melatonin‐pretreated mitochondria effectively suppressed inflammatory reaction (Figure 3N‐U).

### 3.4 Histopathological assessment of liver parenchyma by 72 hours after liver ischaemia‐reperfusion induction

**FIGURE 4:**
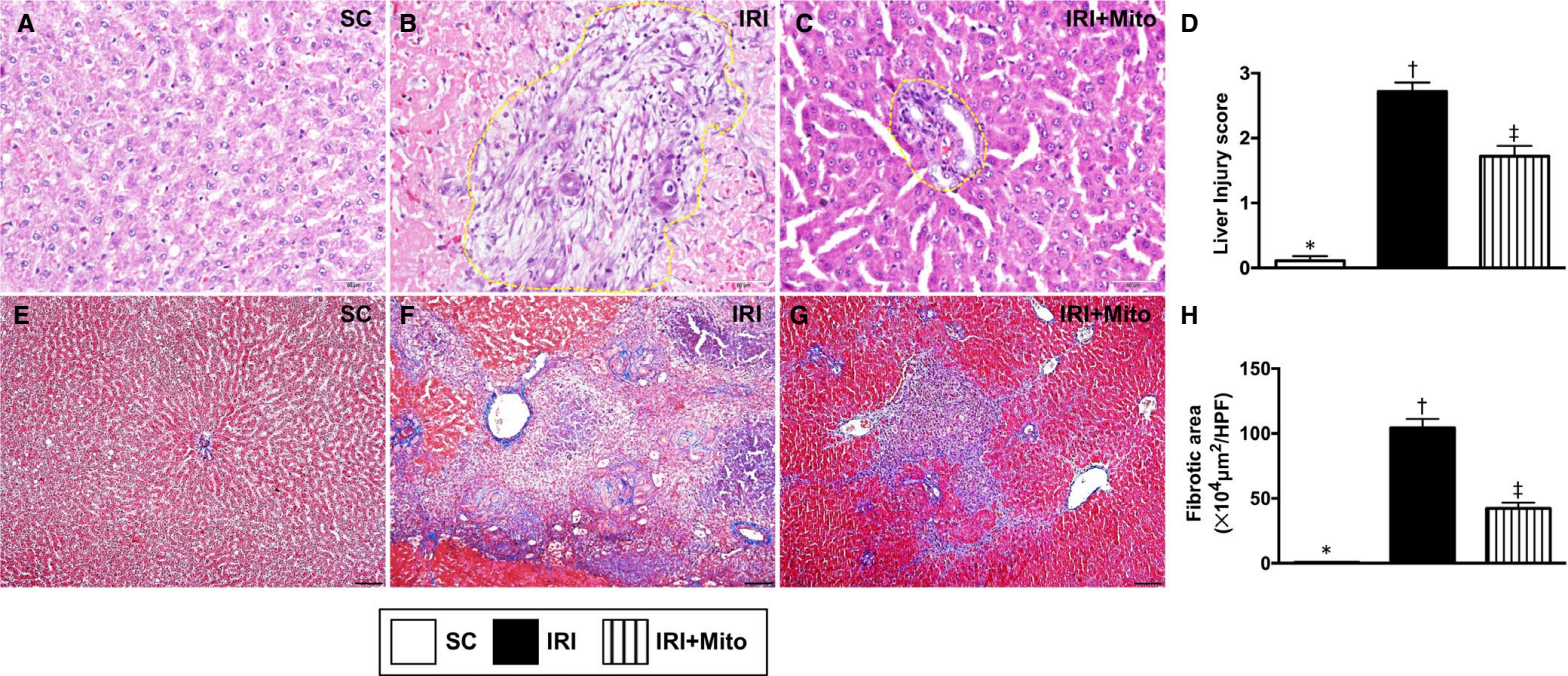
Histopathological assessment of liver parenchyma by 72 h after liver ischaemia‐reperfusion injury induction. A‐C, Illustrating microscopic finding (200×) of haematoxylin and eosin stain for identification of liver injury area (ie ischaemic/infarct area) indicated by dotted lines. D, Analytical result of liver injury score, * vs other groups with different symbols (†, ‡), *P* < 0.0001. E‐G, Illustrating the immunofluorescent microscopic finding (100×) of Masson's trichrome stain for identification of fibrotic area (blue colour). H, Analytical result of liver injury score, * vs other groups with different symbols (†, ‡), *P* < 0.0001. Scale bar in right lower corner represents 100 µm. All statistical analyses were performed by one‐way ANOVA, followed by Bonferroni multiple comparison post hoc test (n = 6 for each group). Symbols (*, †, ‡) indicate significance (at 0.05 level). IRI, ischaemia‐reperfusion injury; Mito, mitochondria; SC, sham control

To assess whether the therapy of melatonin‐pretreated mitochondria would offer benefit on ameliorating the liver injury score, microscopic finding of haematoxylin and eosin‐stained liver sections was performed in each animal. The result showed that the liver injury score was significantly increased in IRI than in SC and IRI + Mito and significantly increased in IRI + Mito than in SC. Additionally, the Masson's trichrome stain displayed that the fibrotic area exhibited an identical pattern of liver injury score among the three groups. These findings implied that melatonin‐pretreated mitochondria preserved the liver parenchymal architecture (Figure 4).

### 3.5 Inflammatory cell infiltration in liver parenchyma by 72 hours after liver ischaemia‐reperfusion induction

**FIGURE 5:**
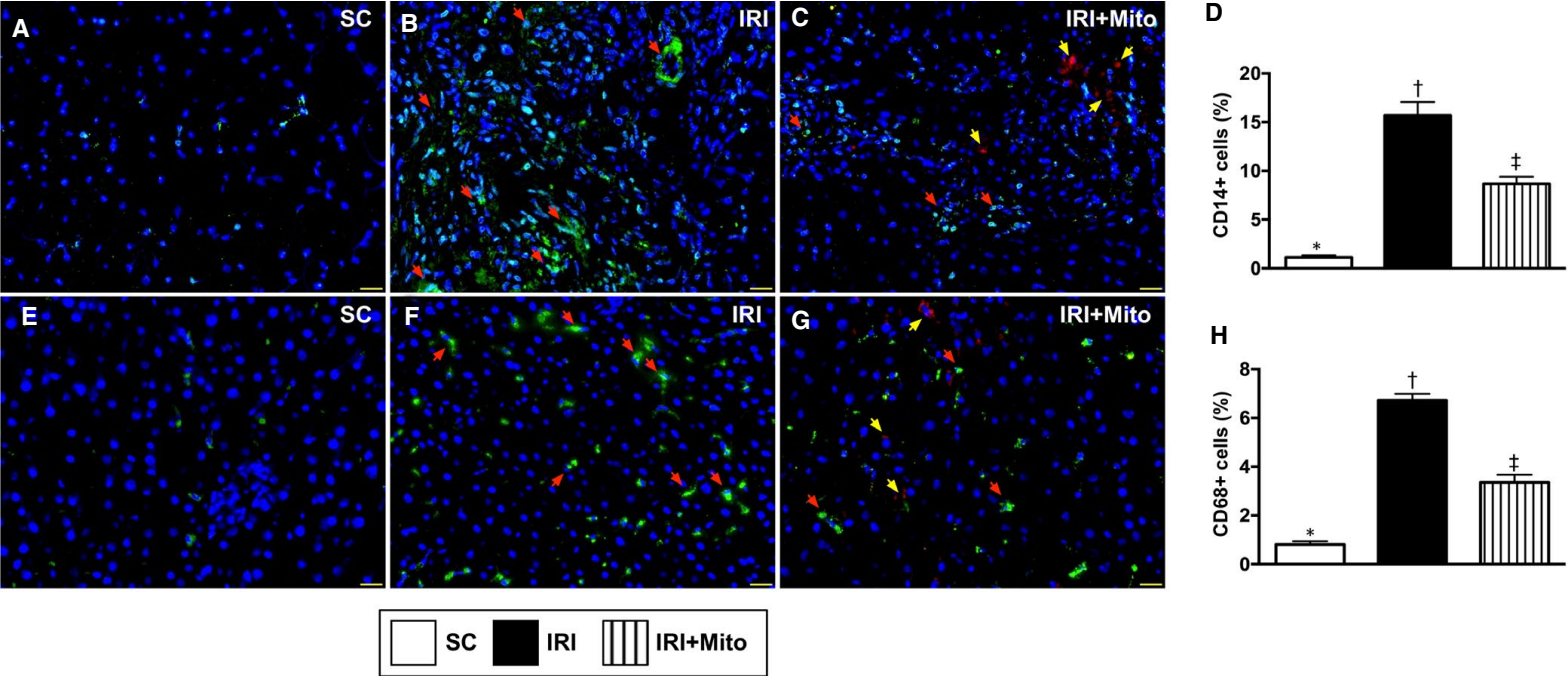
Inflammatory cell infiltration in liver parenchyma by 72 h after liver ischaemia‐reperfusion injury induction. A‐C, Illustrating the immunofluorescent (IF) microscopic finding (400×) for identification of CD14^+^ cells (ie green colour indicated by red arrows) in liver parenchyma. D, Analytical result of number of positively stained CD14 cells, * vs other groups with different symbols (†, ‡), *P* < 0.0001. E‐G, Illustrating the IF microscopic finding (400×) for identification of CD68 cells (ie green colour indicated by red arrows) in liver parenchyma. H, Analytical result of number of positively stained CD68 cells, * vs other groups with different symbols (†, ‡), *P* < 0.0001. Red colour in (C) and (G) (yellow arrows) indicated the dye stained exogenous mitochondria. Scale bar in right lower corner represents 100 µm. All statistical analyses were performed by one‐way ANOVA, followed by Bonferroni's multiple comparison post hoc test (n = 6 for each group). Symbols (*, †, ‡) indicate significance (at 0.05 level). IRI, ischaemia‐reperfusion injury; Mito, mitochondria; SC, sham control

To examine the cellular level of inflammatory cell infiltrations in liver parenchyma, the IF microscope was performed. Consistent with the protein expressions of inflammatory biomarkers, the IF microscope demonstrated that the CD14^+^ and CD68^+^ cells in liver parenchyma, two indicators of the inflammation, were significantly increased in IRI than in SC. Both parameters were significantly reversed in IRI animals after receiving melatonin‐pretreated Mito therapy (Figure 5).

### 3.6 Cellular expressions of γ‐H2AX and MMP‐9 in liver parenchyma by 72 hours after liver ischaemia‐reperfusion induction

**FIGURE 6:**
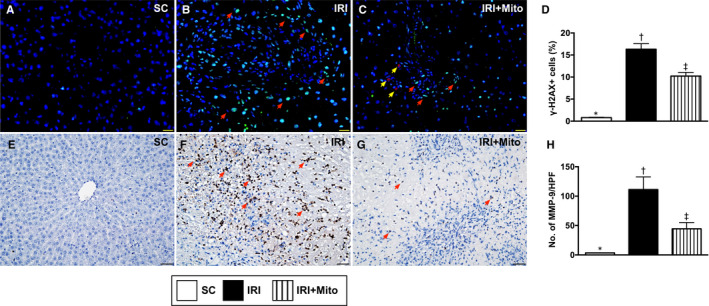
Cellular expressions of γ‐H2AX and MMP‐9 in liver parenchyma by 72 h after liver ischaemia‐reperfusion injury induction. A‐C, Illustrating the immunofluorescent microscopic finding (400×) for identification of cellular expressions of γ‐H2AX in liver ischaemic area (ie green colour indicated by red arrows). D, Analytical result of number of γ‐H2AX+ cells, * vs other groups with different symbols (†, ‡), *P* < 0.0001. Red colour in (C) (yellow arrows) indicated the dye stained exogenous mitochondria. Scale bar in right lower corner represents 20 µm. E‐G, Illustrating the microscopic finding of immunohistochemical stain for identification of matrix metalloproteinase (MMP)‐9 (brown colour indicated by red arrows). H, Analytical result of number of MMP‐9+ cells per high‐power field, * vs other groups with different symbols (†, ‡), *P* < 0.0001. HPF, high‐power field. Scale bar in right lower corner represents 50 µm. All statistical analyses were performed by one‐way ANOVA, followed by Bonferroni's multiple comparison post hoc test (n = 6 for each group). Symbols (*, †, ‡) indicate significance (at 0.05 level). IRI, ischaemia‐reperfusion injury; Mito, mitochondria; SC, sham control

Finally, we found that the cellular expressions of γ‐H2AX, an indicator of DNA‐damaged marker, and MMP‐9, an indicator of acute innate inflammatory reaction, were significantly higher in IRI than in SC and IRI + Mito, and significantly higher in IRI + Mito than in SC (Figure 6).

## 4 DISCUSSION

This study which investigated the impact of melatonin‐pretreated Mito on rat acute liver IR yielded several striking preclinical implications. First, the novel finding was that the ^31^P‐MRS technique was able to clearly identify the amount of energy storage (ie hepatic levels of ATP) and energy activity (ie NADH indicated the ATP metabolic rate) in hepatocytes of living animals. Second, exogenous Mito supply was able to enrich hepatocyte Mito to avoid exhausting the mitochondrial ATP in the hepatocytes during the IRI. Third, melatonin‐pretreated mitochondrial therapy effectively protects the liver from acute IRI mainly through inhibiting the generation of oxidative stress, inflammation, apoptosis, fibrosis and DNA damage.

As a non‐invasive and accurate instrument, the advantage of utilizing hepatic ^31^P‐MRS for detecting the metabolism of different liver disease entities has been extensively discussed.^39^, ^40^, ^41^ However, there is still lacking data to address the situation of ATP metabolism and consumption in hepatocytes in setting of IRI with the exogenous mitochondrial supply. Additionally, a non‐invasive tool with precise diagnostic feature is in urgent need for our daily clinical practice to identify the degree of ATP consumption and depletion in setting of IRI, especially in acute liver IRI, has not yet been reported. These issues encourage us to carry out this experimental study. One novel finding in the present study was that the ^31^P‐MRS tool clearly identified that the level of ATP in hepatocytes of living animals was remarkably reduced in IRI animals, suggesting that the mitochondria/ATP, indicator of energy, was significantly depleted in hepatocytes. However, this parameter was remarkably reversed in IRI animals treated by melatonin‐pretreated Mito. Accordingly, our findings, in addition to extending the findings of previous studies,^39^, ^40^, ^41^ pinpoint the exciting potential of studying metabolic processes in the human liver in vivo.

Accurate diagnosis of disease is of course very important, and the effective treatment of the disease is no doubted essential. Plentiful data have shown that exogenous mitochondrial transfusion not only effectively rescued the function and integrity of endogenous mitochondria but also refreshed the depleted mitochondria in organ, resulting in preserving the ischaemia‐reperfusion‐related organ dysfunction.^16^, ^46^ The most important finding in the present study was that melatonin‐pretreated exogenous mitochondria not only refreshed the mitochondria (ie increased ATP) in hepatocytes but also attenuated the anatomical‐histopathological changes of liver parenchyma (ie ameliorated the liver injury score and fibrosis) and circulatory level of AST and ALT (ie two indices of liver function/hepatocyte damage). Our findings, in addition to strengthening the findings of previous studies,^16^, ^46^ encourage us to initiate the clinical trial for consideration of exogenous mitochondrial transfusion for acute liver IRI patients, especially in those refractory to conventional treatment.

Plentiful studies have previously established that IRI always elicits inflammatory reaction, DNA damage, apoptosis and generation of oxidative stress, resulting in organ damage, tissue necrosis and unfavourable outcomes.^45^, ^46^, ^47^, ^48^, ^49^ Additionally, increased oxidative stress in circulation and intracellular/mitochondrial compartment notably down‐regulated mitochondrial function and depleted energy‐storage capacity.^16^, ^46^ An essential finding in the present study was that these above‐mentioned parameters of molecular‐cellular perturbations^45^, ^46^, ^47^, ^48^, ^49^ were substantially increased in IRI animals as compared to SC animals. In this way, our findings, in addition to being consistent with the findings from previous studies,^45^, ^46^, ^47^, ^48^, ^49^ could, once again, explain why the liver enzyme, fibrosis and liver injury score were markedly increased in IRI animals. Of importance was that melatonin‐pretreated Mito therapy significantly suppressed inflammatory reaction, DNA damage, oxidative stress, cellular apoptosis and fibrosis.

**FIGURE 7:**
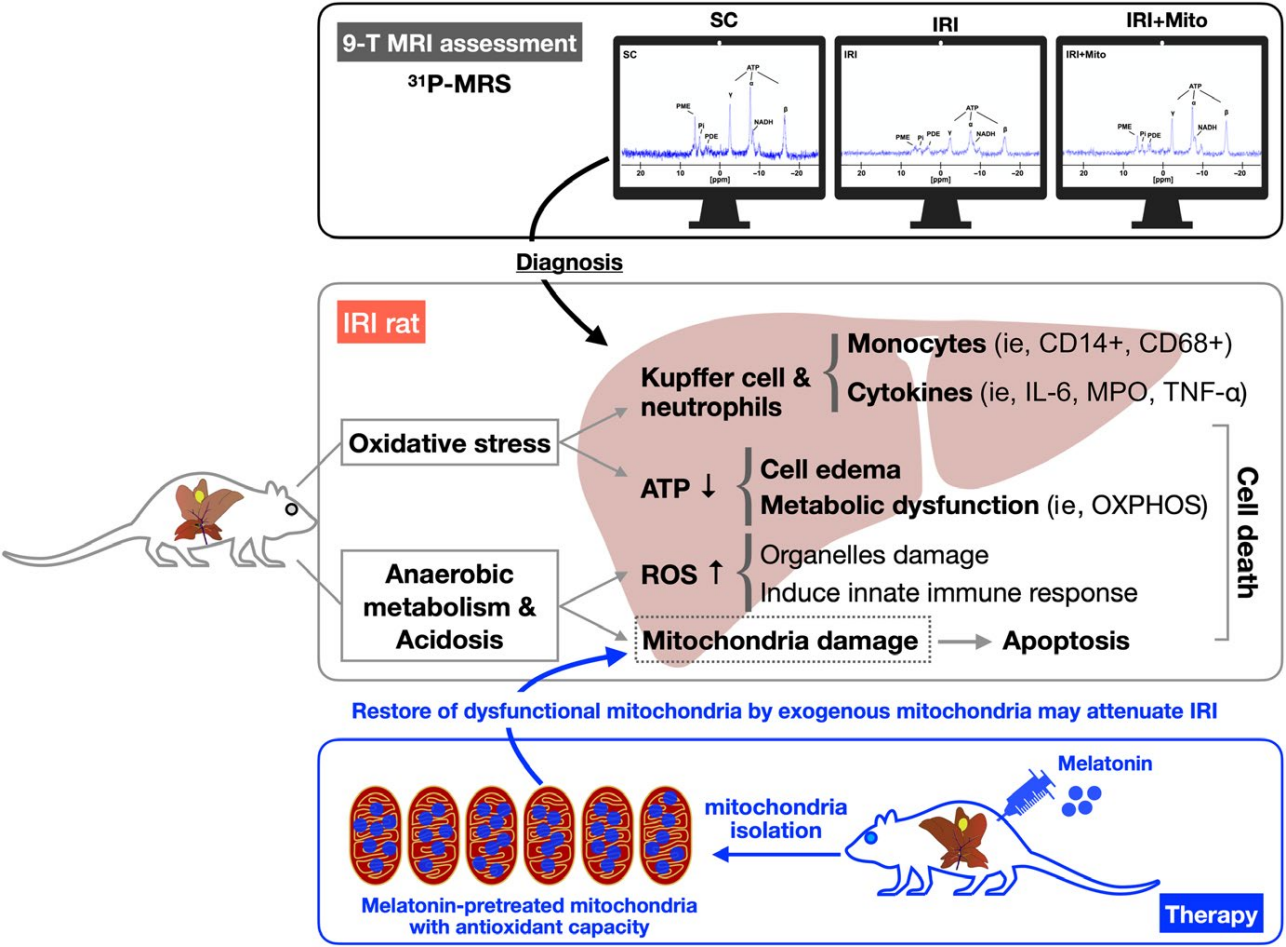
Schematically proposed mechanism of mitochondrial transfusion for reducing acute liver ischaemia‐reperfusion injury in rat. ^31^P‐MRS, ^31^P‐magnetic resonance spectroscopy; ATP, adenosine triphosphate; IL‐6, interleukin 6; IRI, ischaemia‐reperfusion injury; Mito, mitochondria; MPO, myeloperoxidase; ROS, reactive oxygen species; SC, sham control; TNF‐α, tumour necrosis factor alpha

Finally, Figure 7 schematically summarized the innovative findings of our study to facilitate readers’ understanding. Conclusively, we first developed the ^31^P‐MRS technique for precise diagnosis of ATP energy metabolism in the liver. Second, we created an animal model of acute liver IRI and measured the alternations of molecular‐cellular perturbations in liver parenchyma and circulation as well as identified the ultrastructural changes of liver parenchyma. Finally, we clearly proved that melatonin‐pretreated exogenous mitochondria effectively protected the liver against IRI mainly through refreshment of the endogenous mitochondria which were at the gate of death in IRI hepatocytes.

It is well known that melatonin is a dietary supplement that is very safe and non‐toxic to humankind. Some recent data have even shown that melatonin therapy effectively protected the lung against COVID‐19 infection mainly through regulating the immune system, that is immunomodulation.^50^, ^51^, ^52^, ^53^ These mentioned issues^50^, ^51^, ^52^, ^53^ and the results of our study raise the need of consideration melatonin could be utilized for those of acute liver IRI patients shortly, especially in those of patients who are refractory to the conventional treatment.

### 4.1 Study limitation

This study has limitations. First, although the results were attractive and promising, study period was relatively short (ie the study period was only 72 hours). Accordingly, the long‐term impact of melatonin‐pretreated Mito therapy on protecting the liver against IRI is still currently unclear. Second, despite the impact of melatonin on suppressing the cellular infiltration was clearly clarified, the therapeutic impact of this regimen on regulating the macrophage polarization (ie M1/M2 ratio) was not investigated. Third, although extensive works were accomplished in the present study, the exact underlying mechanisms of melatonin‐pretreated Mito therapy on protecting the liver against IRI remain regrettably uncertain. Accordingly, based on our results, the proposed schematic mechanism of melatonin‐pretreated Mito therapy on safeguarding the liver in setting of IRI only was illustrated in Figure 7.

In conclusion, ^31^P‐MRS provided a reliable method for elucidating the level, activity and metabolic rate of ATP in hepatocytes of living animals. Besides, the results of the present study demonstrated that melatonin‐pretreated Mito therapy effectively protected the liver against IRI in rat.

## CONFLICT OF INTEREST

The authors confirm that there are no conflicts of interest.

## AUTHOR CONTRIBUTIONS

**Sheung‐Fat Ko:** Conceptualization (equal); Data curation (equal); Project administration (lead); Writing‐review & editing (equal). **Yi‐Ling Chen:** Conceptualization (equal); Formal analysis (equal); Investigation (equal); Writing‐original draft (equal). **Pei‐Hsun Sung:** Formal analysis (equal); Investigation (equal). **John Y. Chiang:** Writing‐review & editing (equal). **Yi‐Ching Chu:** Formal analysis; Investigation. **Chung‐Cheng Huang:** Formal analysis (equal); Investigation (equal). **Chi‐Ruei Huang:** Formal analysis; Investigation. **Hon‐Kan Yip:** Conceptualization; Data curation; Writing‐original draft (equal); Writing‐review & editing (equal).

## Data Availability

The data that support the findings of this study are available from the corresponding author upon reasonable request.
